# Pulmonary exposure to carbon black nanoparticles and vascular effects

**DOI:** 10.1186/1743-8977-7-33

**Published:** 2010-11-05

**Authors:** Lise K Vesterdal, Janne K Folkmann, Nicklas R Jacobsen, Majid Sheykhzade, Håkan Wallin, Steffen Loft, Peter Møller

**Affiliations:** 1 Department of Public Health, Section of Environmental Health, University of Copenhagen, Copenhagen, Denmark; 2 National Research Centre for the Working Environment, Lersø Park Allé 105, 2100 Copenhagen, Denmark; 3 Department of Pharmacology and Pharmacotherapy, Faculty of Pharmaceutical Sciences, University of Copenhagen, Copenhagen, Denmark

## Abstract

**Background:**

Exposure to small size particulates is regarded as a risk factor for cardiovascular diseases.

**Methods:**

We exposed young and aged apolipoprotein E knockout mice (*apoE^-/-^*) to carbon black (Printex 90, 14 nm) by intratracheal instillation, with different dosing and timing, and measured vasomotor function, progression of atherosclerotic plaques, and VCAM-1, ICAM-1, and 3-nitrotyrosine in blood vessels. The mRNA expression of *VCAM-1*, *ICAM-1*, *HO-1*, and *MCP-1 *was examined in lung tissue.

**Results:**

Young *apoE^-/- ^*mice exposed to two consecutive 0.5 mg/kg doses of carbon black exhibited lower acetylcholine-induced vasorelaxation in aorta segments mounted in myographs, whereas single doses of 0.05-2.7 mg/kg produced no such effects. The phenylephrine-dependent vasocontraction response was shifted toward a lower responsiveness in the mice exposed once to a low dose for 24 hours. No effects were seen on the progression of atherosclerotic plaques in the aged *apoE^-/- ^*mice or on the expression of VCAM-1 and ICAM-1 and the presence of 3-nitrotyrosine in the vascular tissue of either young or aged *apoE^-/- ^*mice. The expression of *MCP-1 *mRNA was increased in the lungs of young *apoE^-/- ^*mice exposed to 0.9-2.7 mg/kg carbon black for 24 hours and of aged *apoE^-/- ^*mice exposed to two consecutive 0.5 mg/kg doses of carbon black seven and five weeks prior to sacrifice.

**Conclusion:**

Exposure to nano-sized carbon black particles is associated with modest vasomotor impairment, which is associated neither with nitrosative stress nor with any obvious increases in the expression of cell adhesion proteins on endothelial cells or in plaque progression. Evidence of pulmonary inflammation was observed, but only in animals exposed to higher doses.

## Background

The development of nanotechnology has increased attention to the possible toxicological effects of nanoparticles. Among the many possible effects, cardiovascular outcomes are considered important, because exposure to ambient particulate matter elevates the risk of cardiovascular disease and mortality in epidemiological studies [1-4]. Even though nanoparticles only account for a small volume of the total mass of particles [1], it is believed that their large surface area and number concentration make them an important contributor to these health effects [5,6]. Particulate matter in the small size range have previously been shown to induce altered activity of the fibrinolytic system, a procoagulant state, and endothelial dysfunction and cardiac effects [6-8]. Hallmarks of initial events in atherogenesis include the expression of cell adhesion molecules on the surface of the endothelium, inflammation, and endothelial dysfunction [9,10]. Endothelial dysfunction is an early sign of atherosclerosis [11] and it is believed to be an event that leads to atherosclerosis as well as being a marker of the severity of it [9]. The endothelium plays an important role in maintaining the vascular homeostasis by producing vasoactive factors, that regulate the tone of the vascular system in response to cell surface receptor stimulation or mechanical stress [12]. Moreover, the endothelium is an important factor in the regulation of vascular inflammation and thrombosis [11]. In experimental settings, the vasomotor function can be assessed as endothelial dependent (acetylcholine) or independent (sodium nitroprusside) vasorelaxation or as receptor dependent vasocontraction (phenylephrine). In apolipoprotein E knockout mice (*apoE^-/-^*), which are deficient in apolipoprotein E and represent a model for atherosclerosis in humans, vasomotor function is inversely correlated with plaque-size, whereas it is not affected by hypercholesterolemia [13]. It has been shown that in *apoE*^-/- ^mice, the acetylcholine-induced vasorelaxation was decreased and the phenylephrine-induced maximal vasocontraction was enhanced after 5 months inhalation exposure to concentrated air pollution particles [14,15]. A number of studies have shown acute effects of particle exposure on vasomotor function. For instance, ultrafine TiO_2 _particles were more potent in inducing systemic microvascular dysfunction in rats than fine TiO_2 _particles after inhalation of doses with minimal pulmonary effects [16]. Furthermore, we have shown that systemic exposure to diesel exhaust particles or C_60 _fullerenes by intraperitoneal injection reduced acetylcholine-elicited vasorelaxation in *apoE*^-/- ^mice with a mild degree of atherosclerosis, whereas opposite effects were seen in wild type mice [17,18]. Research concerning the effects of nanosized carbon black (CB) particles on vasomotor function is, however, still very sparse. Nanosized CB is widely used as a component in rubber and as a pigment in paints and inks [19] and it is therefore considered likely that people employed in the manufacture of these product are exposed to nanosized CB. Most CB particles including Printex 90 consist of pure carbon and do not contain adhered polycyclic aromatic hydrocarbons as for instance DEP [20,21]. A number of studies show that airway exposure to concentrated ambient particles and single wall carbon nanotubes promotes the progression of atherosclerosis in *apoE*^-/- ^mice [22-24]. Similarly, pulmonary exposure to nano-sized CB is associated with accelerated plaque area development in the aorta of low-density lipoprotein receptor knockout mice which are also predisposed to develop atherosclerosis [25]. In addition, studies investigating the effects of exposure to particulate matter in the form of air pollution or diesel exhaust particles have shown an increased area of atherosclerotic plaques in the aorta of Watanabe heritable hyperlipidemic rabbits [26,27].

The purpose of the present study was to investigate the concentration-response and timing of effects of single and repeated intratracheal (i.t.) instillations with CB nanoparticles at different time points after exposure of *apoE*^-/- ^mice, which exhibited either a mild or a more severe degree of atherosclerosis. We chose Printex 90 CB which is very well characterised, widely studied for other endpoints than vascular function and may serve as benchmark for carbon based nanoparticles with environmental and occupational health relevance. The effects on vasomotor function were assessed in young *apoE^-/- ^*mice (11-13 weeks) as we have previously shown that the vasomotor function was severely impaired in aged apoE^-/- ^mice [18]. The effects on progression of atherosclerosis were assessed in aged *apoE^-/- ^*mice (48-49 weeks) in which a substantial amount of plaques had developed. We assessed the expression of vascular adhesion molecule (VCAM-1) and intercellular adhesion molecule (ICAM-1) on the endothelium as well as the presence of 3-nitrotyrosine in the vascular tissue. VCAM-1 and ICAM-1 are markers of the immune cell recruitment which is an early event in atherosclerotic plaque formation [28,29] and 3-nitrotyrosine is a biomarker of peroxynitrite [30]. Moreover, the gene expression of *VCAM-1 *and *ICAM-1 *as well as macrophage/monocyte chemoattractant protein 1 (*MCP-1*) and heme oxygenase 1 (*HO-1*) was assessed in lung tissue. *MCP-1 *and *HO-1 *were assessed as markers of inflammation and oxidative stress, respectively.

## Results

### Effect of carbon black on the vasomotor function in young apoE^-/- ^mice

#### Endothelium-dependent vasorelaxation

**Figure 1:**
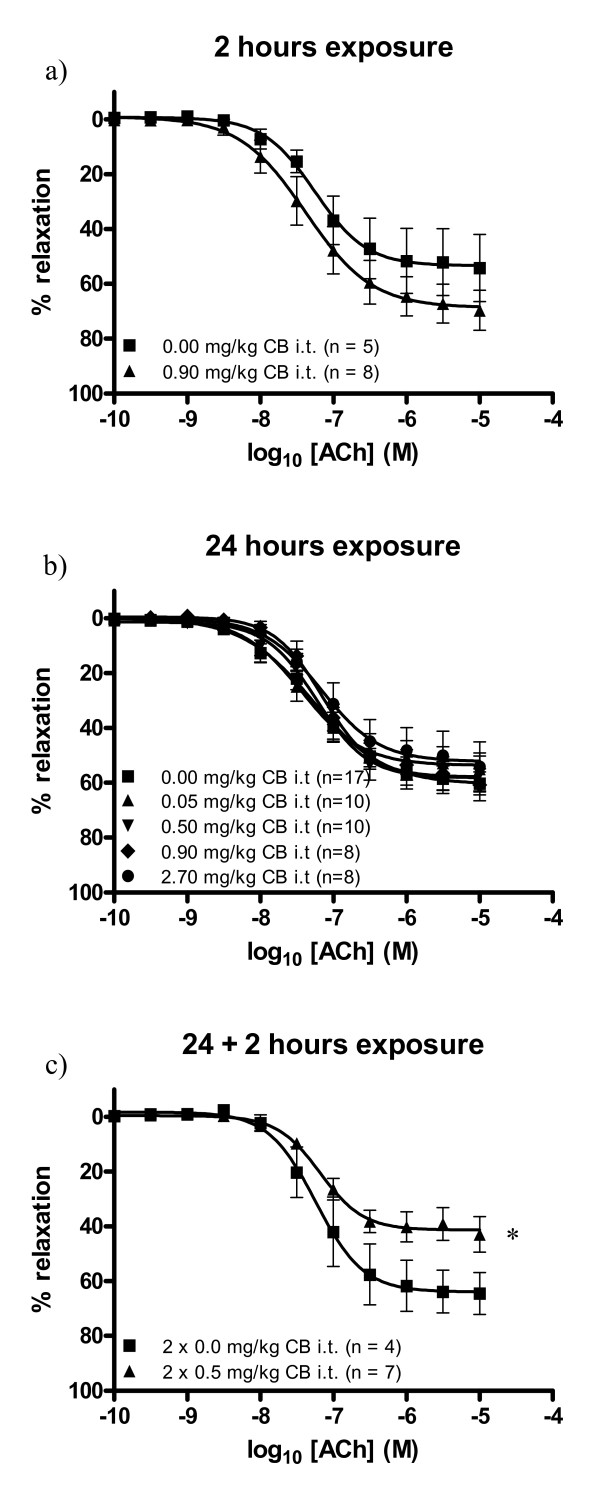
**Endothelium-dependent vasorelaxation of aorta segments from 11-13 weeks old *apoE^-/- ^*mice exposed to carbon black by i.t. instillation**. The response is expressed as the % relaxation of the precontraction tension produced by prostaglandin F_2_α. Each point on the curves represents the cumulative response at each concentration of acetylcholine (ACh). The data are expressed as the mean and SEM (see graph for n-values). * denotes a significant effect on E_max _compared to the control group (P < 0.05, ANOVA with unequal variance between groups).

The group of mice which received two doses of 0.5 mg/kg CB exhibited acetylcholine-dependent vasorelaxation with an E_max _value of 41.3% (95% CI: 37.0-45.5%). This was significantly lower than the E_max _value for the control group which was 63.9% (95% CI: 55.5-72.3%), (Table 1, Figure 1c). No effect of CB exposure was observed in the groups of mice which received a single dose of particles 2 hours or 24 hours prior to sacrifice (Table 1, Figure 1a,b).

**Table 1:** EC_50 _and E_max _values of concentration-response curves

Exposure to carbon black	EC_50 _(nM)			E_max _(%)		
		
	ACh	SNP	PE	ACh	SNP	PE
**2 hours**						
0.0 mg/kg	**55.7 **(24.5-127.1)	**17.5 **(14.4-21.2)	**31.6 **(24.7-40.3)	**53.4 **(43.9-62.9)	**90.4 **(87.5-93.3)	**67.8 **(64.3-71.3)
0.9 mg/kg	**41.6 **(22.0-78.7)	**12.8 **(10.0-16.4)	**21.4 **(18.2-25.2)	**68.7 **(60.2-77.2)	**91.4 **(87.7-95.0)	**70.0 **(67.6-72.4)

**24 hours**						
0.0 mg/kg	**52.5 **(33.5-82.3)	**11.1 **(8.0-15.5)	**8.9 **(7.6-10.4)	**60.5 **(55.1-65.9)	**89.2 **(84.9-93.5)	**73.1 **(71.0-75.2)
0.05 mg/kg	**37.1 **(20.9-65.8)	**7.7 **(5.7-10.3)	**5.5 **(4.3-7.0)*	**53.6 **(47.7-59.5)	**93.9 **(90.1-97.6)	**73.4 **(70.2-76.6)
0.5 mg/kg	**59.0 **(37.3-93.4)	**16.8 **(13.1-21.4)	**5.8 **(5.2-6.5)*	**58.2 **(52.5-63.8)	**89.5 **(85.9-93.0)	**72.0 **(70.3-73.7)
0.9 mg/kg	**71.3 **(47.8-106.1)	**15.7 **(10.5-23.7)	**13.8 **(9.8-19.5)	**57.8 **(52.6-63.0)	**81.9 **(76.5-87.3)	**69.2 **(64.6-73.8)
2.7 mg/kg	**68.4 **(33.2-141.0)	**15.1 **(7.4-30.5)	**13.1 **(8.7-19.8)	**52.2 **(44.1-60.3)	**83.9 **(75.0-92.9)	**70.4 **(65.2-75.7)
	
**24 + 2 hours**						
2 × 0.0 mg/kg	**57.4 **(31.9-103.1)	**12.6 **(8.5-18.8)	**13.6 **(11.2-16.5)	**63.9 **(55.5-72.3)	**90.1 **(84.7-95.5)	**75.9 **(72.8-78.9)
2 × 0.5 mg/kg	**65.8 **(42.3-102.4)	**14.3 **(11.5-17.7)	**17.0 **(14.3-20.3)	**41.3 **(37.0-45.5)*	**91.9 **(88.8-95.1)	**75.1 **(72.4-77.8)

The concentration-response curves were performed for acetylcholine (ACh), sodium nitroprusside (SNP), and phenylephrine (PE) on aorta from 11-13 weeks old female ApoE^-/- ^mice exposed in vivo to 0.0 or 0.9 mg/kg carbon black i.t. 2 hours before sacrifice, to 0; 0.05; 0.5; 0.9 or 2.7 mg/kg carbon black i.t. 24 hours before sacrifice or twice to 0.0 or 0.5 mg/kg carbon black 24 + 2 hours before sacrifice. The data are expressed as means and 95% CI. The number of animals in each group varied. * denotes statistically significant effects on EC_50 _or E_max _as compared to the effect in the control group (P < 0.05, ANOVA with unequal variance between groups).

We found no significant correlation between the relative precontraction tension and the maximal acetylcholine-induced vasorelaxation (Spearman, r = -0.21, P = 0.11). This indicates that the observed decrease in acetylcholine-induced vasorelaxation in the CB exposed mice cannot be attributed to differences in relative precontraction tension.

#### Endothelium-independent vasorelaxation

**Figure 2:**
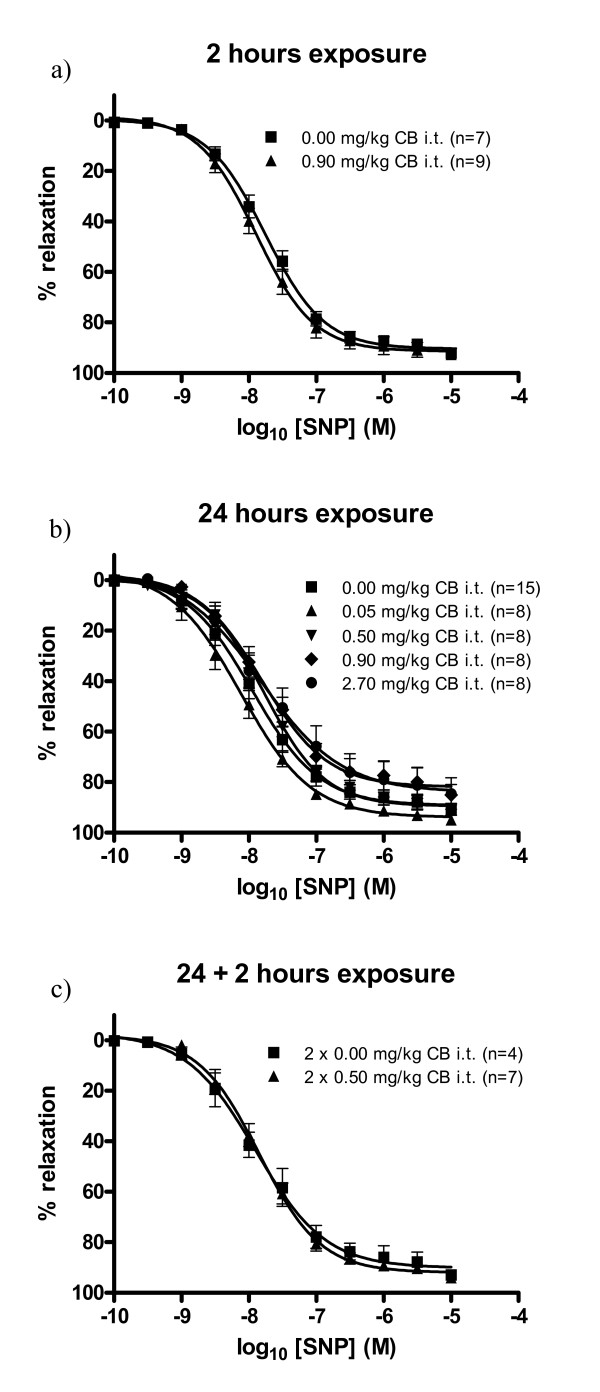
**Endothelium-independent relaxation of aorta segments from 11-13 weeks old *apoE^-/- ^*mice exposed to carbon black by i.t. instillation**. The response is expressed as the % relaxation of the precontraction tension produced by PGF_2_α. Each point on the curve represents the cumulative response at each concentration of sodium nitroprusside (SNP). The data are expressed as the mean and SEM (see graph for n-values).

There were no significant effects of CB exposure on the sodium nitroprusside-induced vasorelaxation in any of the groups of *apoE*^-/- ^mice (Table 1, Figure 2a-c).

#### Receptor-dependent vasocontraction

**Figure 3:**
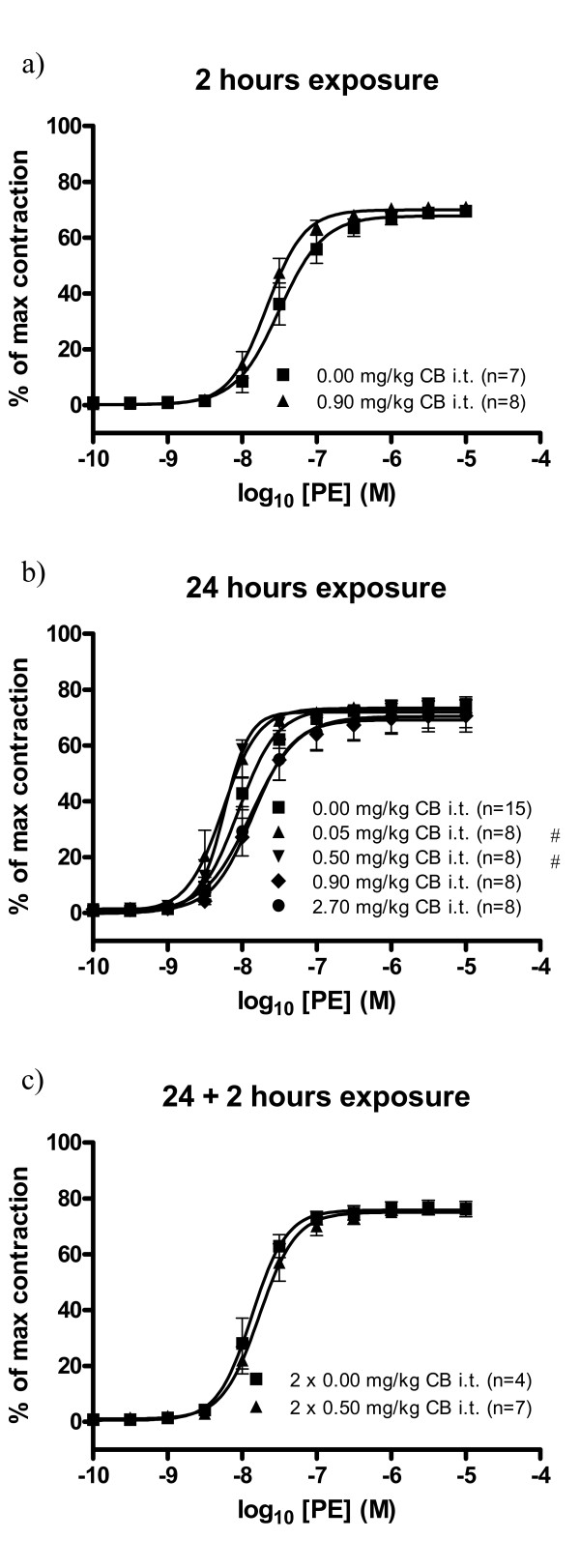
**Receptor-dependent vasocontraction of aorta segments 11-13 weeks old *apoE^-/- ^*mice exposed to carbon black by i.t. instillation**. The response is expressed as the % of the maximal contraction induced by stimulation with a cocktail of K^+^-PSS, PGF_2_α, and U-46619. Each point on the curve represents the cumulative response at each concentration of phenylephrine (PE). The data are expressed as the mean and SEM (see graph for n-values). # denotes a significant effect on EC_50 _compared to the control group (P < 0.05, ANOVA with unequal variance between groups).

The groups of mice which received a single dose of 0.05 or 0.5 mg/kg CB 24 hours prior to sacrifice exhibited phenylephrine-induced vasocontraction with EC_50 _values of 5.5 nM (95% CI: 4.3-7.0 nM) and 5.8 nM (95% CI: 5.2-6.5 nM), respectively. In contrast, the EC_50 _value of the control group was 8.9 nM (95% CI: 7.6-10.4 nM). This indicates that the exposure to the low doses induced a small, but significant, shift in the EC_50 _values towards an increased responsiveness (Table 1, Figure 3b). This was not the case for the groups of mice which received 0.9 or 2.7 mg/kg CB 24 hours prior to sacrifice, as the EC_50 _values seemed to exhibit a slight shift towards a decreased responsiveness. This shift, however, was not statistically significant (Table 1, Figure 3b). No effect of CB exposure was observed in the groups of mice which received a single dose of 0.9 mg/kg 2 hours prior to sacrifice or two doses of 0.5 mg/kg 24 + 2 hours prior to sacrifice (Table 1, Figure 3a,c).

### Effect of carbon black on the progression of atherosclerotic plaques in aged apoE^-/- ^mice

**Figure 4:**
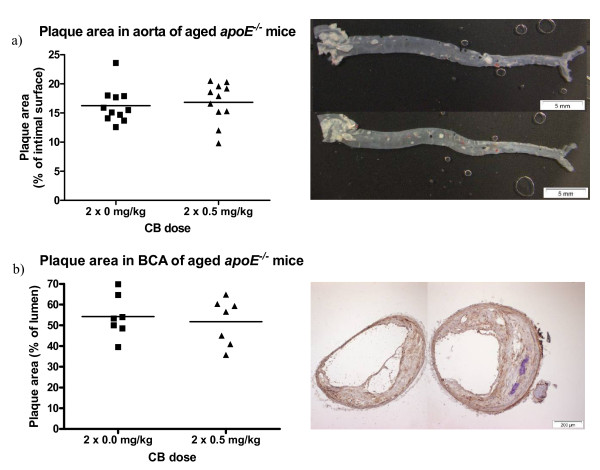
**Progression of atherosclerotic plaques in a) aorta or b) brachiocephalic arteries (BCA) from 48-49 weeks old *apoE^-/- ^*mice exposed to carbon black by i.t. instillation**. In a) plaque area is expressed as % of intimal surface of aorta covered in plaques (left). The means are represented by horizontal lines (n = 11); each symbol represent the result from one animal. On the right, images are shown of the intimal surface of the aorta from the ascending aorta to the femoral arteries representative of the mean plaque areas in the control group (upper) and the exposed group (lower). The atherosclerotic plaques are discernible as white areas on the light grey background of normal aorta. In b) plaque area is expressed as % of lumen of BCA occupied by plaques (left). The means are represented by horizontal lines (n = 7); each symbol represent the result from one animal. On the right, images are shown of BCA sections representative of the means in the control group (left) and the exposed group (right).

Plaque area was determined on the intimal surface of whole aorta of aged mice which received two doses of 0.5 mg/kg CB or vehicle 7 and 5 weeks prior to sacrifice. There was no significant difference in plaque area between the CB exposed group and the control group (Figure 4a). Correspondingly, the plaque area determined in the cross sections of the BCA from the same mice did not show any difference between the CB exposed group and the control group (Figure 4b).

### Effect of carbon black exposure on the expression of surface proteins and the presence of 3-nitrotyrosine in aorta of young apoE^-/- ^mice and in the brachiocephalic artery of aged apoE^-/- ^mice

**Figure 5:**
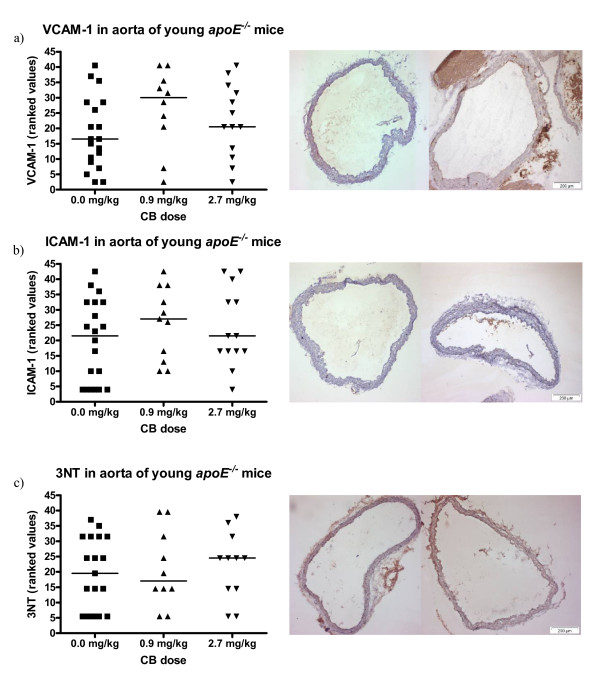
**Expression of VCAM-1 and ICAM-1 on the endothelium and the presence of 3-nitrotyrosine (3NT) in the vascular tissue of aorta from 11-13 weeks old *apoE^-/- ^*mice exposed to carbon black by i.t. instillation**. The antibodies used were HRP-streptavidin conjugated and visualized with DAB. On the left, the data are depicted as geometric means of ranked values representing the amount of staining assessed in 4 sections of aorta from each animal. The medians are depicted as horizontal lines (n = 10-20); each symbol represent the result from one animal. On the right, images are shown of aorta sections representative of the median in the control group (left) and the 2.7 mg/kg exposed group (right). Binding of antibodies can be seen as brown staining of the tissue.

VCAM-1, ICAM-1, and 3-nitrotyrosine was visualized by immunohistochemistry in aorta sections of young *apoE*^-/- ^mice exposed to either 0.9 or 2.7 mg/kg CB or vehicle 24 hours prior to sacrifice. No significant effect of CB exposure on the expression of the surface proteins or on the presence of 3-nitrotyrosine could be observed when compared to the control group (Figure 5a-c).

**Figure 6:**
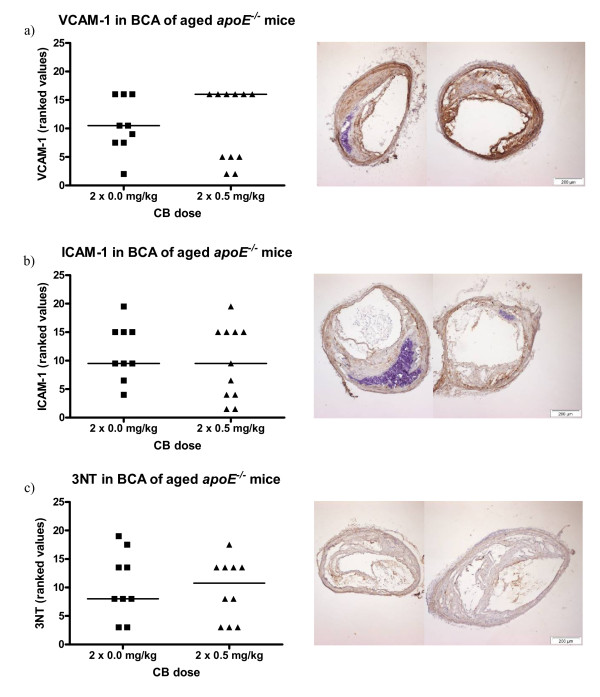
**Expression of VCAM-1 and ICAM-1 on the endothelium and the presence of 3-nitrotyrosine (3NT) in the vascular tissue of brachiocephalic arteries (BCA) from 48-49 weeks old *apoE^-/- ^*mice exposed to carbon black by i.t. instillation**. The antibodies used were HRP-streptavidin conjugated and visualized with DAB. On the left, the data are depicted as geometric means of ranked values representing the amount of staining assessed in 4 sections of aorta from each animal. The medians are represented by horizontal lines (n = 9-11); each symbol represent the result from one animal. On the right, images are shown of BCA sections representative of the medians in the control group (left) and the exposed group (right). Binding of antibodies can be seen as brown staining of the tissue.

VCAM-1, ICAM-1, and 3-nitrotyrosine was also visualized by immunohistochemistry in BCA sections of aged *apoE*^-/- ^mice exposed twice to 0.5 mg/kg CB or vehicle 7 weeks and 5 weeks prior to sacrifice. No effect of CB exposure on the expression of the surface proteins or on the presence of 3-nitrotyrosine could be observed when compared to the control group (Figure 6a-c).

### Effect of carbon black on the expression of mRNA in lung tissue of apoE^-/- ^mice

**Figure 7:**
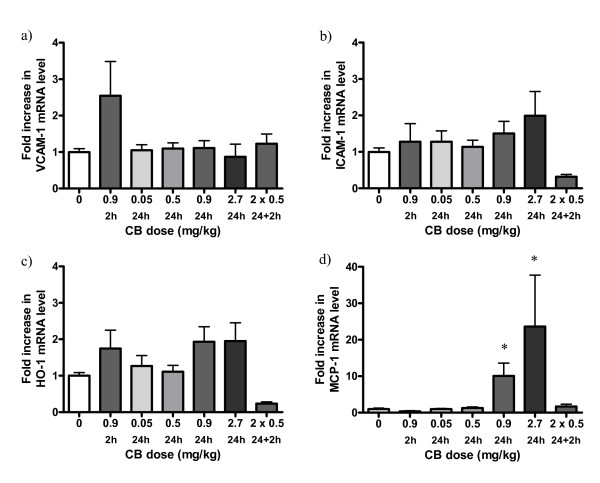
**mRNA expression in lung tissue of 11-13 weeks old *apoE^-/- ^*mice exposed to carbon black by i.t. instillation**. The data are expressed as fold increases in mRNA expression compared to the control group. The number of animals in each group is 22 (0 mg/kg), 5-6 (0.9 mg/kg, 2 h), 10 (0.05 mg/kg, 24 h), 9 (0.5 mg/kg, 24 h), 6 (0.9 mg/kg, 24 h), 4 (2.7 mg/kg, 24 h), 6 (2 × 0.5 mg/kg). * denotes statistically significant differences in mRNA level when compared to the control groups (P < 0.05, Kruskall-Wallis test, post-hoc Tukey-type multiple comparison test).

**Figure 8:**
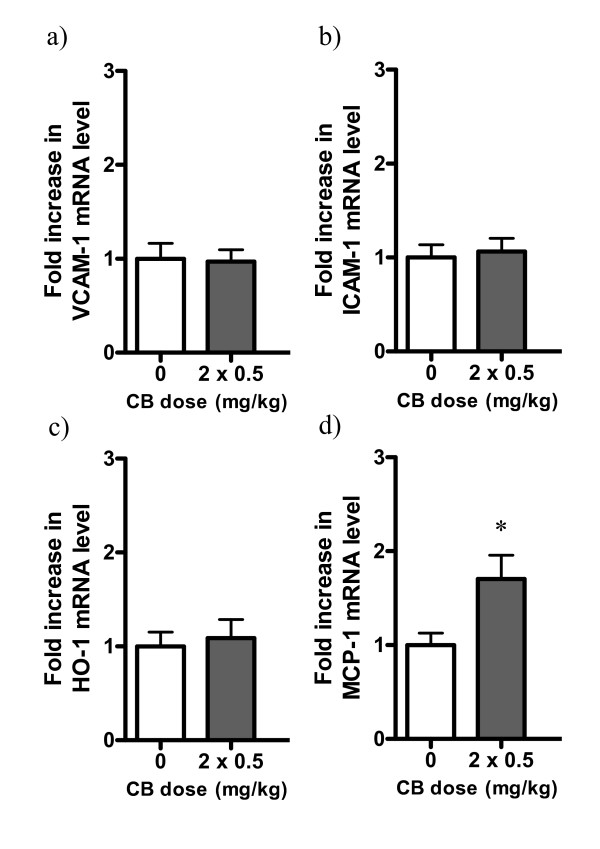
**mRNA expression in lung tissue of 48-49 weeks old *apoE^-/- ^*mice exposed to carbon black by i.t. instillation**. The data are expressed as fold increases in mRNA expression compared to the control group (n = 10-11 in each group). * denotes statistically significant differences in mRNA level when compared to the control groups (P < 0.05, Mann-Whitney *U*-test).

There were no significant differences in the expression of *VCAM-1*, *ICAM-1*, *HO-1 *or *MCP-1 *in the lungs of young *apoE*^-/- ^mice 24 hours after exposure to 0.05 or 0.5 mg/kg CB, when compared with the control group. However, there was a significant increase in the expression of *MCP-1 *24 hours after exposure to 0.9 or 2.7 mg/kg CB, when compared to the control group (Figure 7). There were no significant differences in the expression of any of the four genes in the lungs of young *apoE*^-/- ^mice 2 hours after exposure to 0.9 mg/kg CB or after two consecutive exposures to 0.5 mg/kg CB 24 + 2 hours prior to sacrifice (Figure 7). There was a significant increase in the expression of *MCP-1 *in the lungs of aged *apoE*^-/- ^mice after two consecutive exposures to 0.5 mg/kg CB 7 and 5 weeks prior to sacrifice when compared to the control group (Figure 8).

## Discussion

The results obtained in the present study show an effect on vasomotor function in aorta of *apoE*^-/- ^mice after i.t. instillation with CB. This alteration occurred without overt differences in expression of vascular endothelial cell adhesion proteins and nitrosative stress in the endothelium, but to a certain extent with increases in pulmonary inflammation.

The altered response to acetylcholine-induced vasorelaxation in the particle-exposed group, with unaltered sodium nitroprusside-induced response, suggests that endothelial NO availability, rather than the smooth muscle sensitivity to or extracellular consumption of NO, is affected by the pulmonary exposure to CB. The same type of effect has been observed for TiO_2 _particles [16,31]. This effect could come about by either decreased NO production or quenching of NO by excessive production of reactive oxygen species in the endothelium. In addition, acetylcholine also induces vasorelaxation through other mechanisms (e.g. cyclooxygenase) and future studies are needed to determine these differences. The mechanisms could be pursued in future studies with focus on the NO system and oxidative stress, including animal pretreatment with e.g. eNOS cofactors, anti-inflammatory or antioxidant compounds and addition of similar compounds to vessel segments. Studies of vasomotor function in the aorta of *apoE^-/- ^*mice have shown that the mechanisms of endothelial dysfunction involved depletion of NO by reaction with superoxide anion radicals as well as decay of tetrahydrobiopterin by peroxynitrite [32,33]. The uncoupling of eNOS is characterized by a biochemical vicious circle in which eNOS begins to generate peroxynitrite and then gradually shifts towards only generating superoxide anion radicals [34]. Evidence of eNOS uncoupling has been observed in cardiac tissue of mice exposed to CB by inhalation [35]. There is also evidence that exposures to diesel exhaust and concentrated air pollution particles are associated with eNOS uncoupling in endothelial cells and vasomotor dysfunction [36,37]. Excessive production of peroxynitrite can be assessed as increased production of 3-nitrotyrosine residues on proteins [38]. In the present study no dose-dependent increase in intensity of 3-nitrotyrosine staining in aorta and BCA vessels was observed. It is possible that the duration of the exposure may have been too short to allow sufficient accumulation of 3-nitrotyrosine residues on proteins. In contrast, clear evidence of 3-nitrotyrosine staining as well as accelerated plaque progression was observed in a study of 6 months exposure to concentrated air pollution particles [15].

The decreased endothelium-dependent acetylcholine response was only observed in the *apoE^-/- ^*mice exposed to two successive i.t. instillations of CB, whereas the response was unaffected both 2 hours and 24 hours after a single instillation. This indicates that repeated exposures of small doses of CB are more important than a single large dose in the generation of endothelial impairment in *apoE^-/- ^*mice. Whether this effect might be attributed to a translocation of CB nanoparticles from the lungs to the vascular system is unknown. Translocation of ultrafine particles to the systemic circulation after i.t. instillation has been demonstrated in a number of studies [39,40], although the translocated fraction of the dose is usually less than 1% in studies of single pulmonary exposure to model particles [41]. However, there are a number of variables that may have caused increased translocation of particles in our study: 1) i.t. instillation of CB has been shown to induce lung inflammation and increased alveolar-capillary permeability in wild type mice [42], 2) *apoE^-/- ^*mice have compromised vascular permeability [43,44], 3) lipopolysaccharide-induced pulmonary inflammation has been shown to increase the translocation of subsequent particle exposure [45], 4) i.t. instillation of CB has been associated with a larger inflammatory response in *apoE^-/- ^*mice than in wild type mice [46]. It is thus possible that inflammation of the lungs caused by the first exposure to CB particles might in a similar manner have increased the translocation of the second dose of particles into the bloodstream leading to a significant effect on vasomotor function. Previously, we have shown that i.t. instillation of 18 or 54 μg/mouse of CB in *apoE^-/- ^*mice was associated with increased expression of *MIP-1*, *MCP-1*, and *IL-6 *inflammatory markers in lung tissue after 3 hours and influx of neutrophils in bronchoalveolar fluid 24 hours after the exposure [46]. The *MCP-1 *expression levels were 18 and 38-fold increased in lung tissue 24 hours after the instillation [46]. In the present study, the same doses were associated with 10 and 23-fold increased *MCP-1 *expression levels in the lung tissue. The doses of 18 and 54 μg/mouse correspond to the doses of 0.9 and 2.7 mg/kg used in the present study. Alternatively, pulmonary inflammation has been demonstrated to induce systemic effects such as promoting the development of atherosclerosis [47]. However, in the present study we found increased expression of *MCP-1 *only in the young mice exposed to the two highest doses of CB for 24 hours and not in the mice exposed to the low doses or exposed twice. This suggests that the effects on endothelial function cannot in this case be attributed to pulmonary inflammation.

In contrast to earlier studies on diesel exhaust particles and C_60 _fullerenes [17,18], we did observe altered function of phenylephrine-induced vasocontraction. The effect was a relatively small shift towards an increased responsiveness for the mice exposed for 24 hours to the two lowest doses of CB. However, this effect was not seen for any of the other exposure groups. The observed effect is in agreement with the hypothesis that oxidative stress could have altered the signalling pathways of the cells and decreased the bio-availability of NO. This would increase the ability of the blood vessels to contract. Printex 90 does indeed generate high levels of reactive oxygen species [48-50]. However, we did not detect any increases in *HO-1 *(oxidative stress) in the lung tissue of any of the exposure groups. The vasomotor impairment is therefore unlikely to have been caused by systemic effects secondary to oxidative stress in the lungs.

The expression of adhesion molecules on the surface of endothelial cells is considered a hallmark in atherogenesis where they are localized to sites for plaque formation [51]. VCAM-1 is expressed mainly in plaques and is considered particularly important in early foam cell formation, whereas ICAM-1 is more widely expressed both in plaques and in non-plaque areas [52,53]. Still, we found no convincing evidence of increased gene or protein expression of VCAM-1 and ICAM-1 in the CB exposed animals. Studies on microarray analysis of endothelial cell cultures exposed to CB have shown upregulation of both ICAM-1 and VCAM-1 [54]. Increased ICAM-1 and VCAM-1 expression has also been observed in rabbit aorta tissue after exposure to air pollution particles by i.t. instillation [55]. Another study has shown increased VCAM-1 staining in BCA vessels of *apoE^-/- ^*mice after pulmonary exposure to single-walled carbon nanotubes [56]. Interestingly, both studies used repeated pulmonary exposures and also observed accelerated progression of atherosclerosis. In the present study, the staining for ICAM-1 and VCAM-1 was more pronounced in blood vessels of the aged *apoE^-/- ^*mice compared to the young mice, although it should be emphasized that it was not the same type of blood vessel that was investigated in the two age groups. Still, we take these observations as evidence proving technical ability to detect expression of ICAM-1 and VCAM-1. The unaltered expression of ICAM-1 and VCAM-1 corresponds well with the unaltered *ICAM-1 *and *VCAM-1 *mRNA levels in the CB exposed mice of either age group as well as the unchanged progression of atherosclerosis in the aged CB exposed mice. This suggests that the doses may have been too small to elicit changes in progression of atherosclerosis. However, the aged mice did exhibit a significant increase in *MCP-1 *in the lungs indicating that, 5 weeks after the last CB instillation, pulmonary inflammation persisted. Our original aim was to use the same exposure scenario as Li et al. used in their study of single-walled carbon nanotubes, which was one instillation every other week for eight weeks [56]. However, a number of the *apoE^-/- ^*mice died after each i.t. instillation and the exposures were therefore discontinued after the second instillation, corresponding to 5 and 7 weeks prior to sacrifice. It is possible that pulmonary exposure to CB in younger *apoE^-/- ^*mice on chow or Western diet would have been associated with progression of plaques as accelerated plaque progression has been shown in *LDLr *knockout mice after expose to CB by i.t. dispersion once a week for ten weeks [25].

## Conclusions

Here we show that exposure to CB particles with a primary nano-size diameter is associated with modest vasomotor impairment in dyslipidemic *apoE^-/- ^*mice. Repeated exposures of small doses of CB is more important that a single large dose in the generation of endothelial impairment in *apoE^-/- ^*mice. The vasomotor impairment is associated neither with nitrosative stress nor with obvious increases in expression of cell adhesion proteins on endothelial cells or in plaque progression. Evidence of pulmonary inflammation was observed, but it was only for animals exposed to higher doses. Even though a direct comparison to humans cannot be drawn, these findings could indicate that pulmonary exposure to CB nanoparticles may be associated with vasomotor impairment in susceptible individuals.

## Methods

### Animals

The *apoE*^-/- ^(C57BL/6-Apoe ^tm1^) mice were purchased from Taconic MB (Ejby, DK) at the age of 5-8 weeks and were all female. They were housed in cages with a 12 hour day-night cycle and were provided with unlimited access to standard mouse chow (Standard Altromin no.1314, Lage, DE) and tap water. The *apoE*^-/- ^mice were exposed to CB at the age of 11-13 (young) or 48-49 (aged) weeks. Institutional guidelines for animal welfare were followed and the Danish Ethical Committee for Animal Studies approved the animal experiments.

### Particles

The nanosized CB particles were chosen for this study because they are well characterized and have been used in research of other end points than vascular function. These include inflammation and DNA damage in the lungs of *apoE^-/- ^*mice following instillation [46] as well as apoptosis, cytokine release, and genotoxicity in cell culture [57-59]. The CB (Printex 90) was a gift from Evonik Degussa GmbH (Frankfurt, DE) (primary particle size 14 nm; surface area 300 m^2^/g). For the i.t. instillations, the particles were suspended by sonication in vehicle using a Branson Sonifier S-450D (Branson Ultrasonics Corp., Danbury, CT, USA) equipped with a disruptor horn (Model number: 101-147-037). The vehicle consisted of 90% sterile, isotonic saline and 10% bronchoalveolar lavage fluid. The latter was prepared by flushing the lungs of unexposed *apoE*^-/- ^mice twice with 0.6 ml isotonic saline. The particle suspensions were sonicated on ice. The Branson Sonifier was operated under the following settings: total sonication time 15 min, alternating with a 55 s pulse ON and a 5 s pause and amplitude of 10%. Control solutions were prepared containing 90% isotonic saline and 10% bronchoalveolar fluid from *apoE*^-/- ^mice. The suspensions were divided in aliquots and immediately frozen at -80°C until use. The suspensions were thawed at room temperature prior to use. We have used the same type of suspension of CB in this and an earlier study on pulmonary toxicity of particles in *apoE^-/- ^*mice; the suspension of CB in the instillation solution exhibited a bimodal size-distribution with frequent peaks at 1.2 and 5.5 μm by dynamic light scattering [46]. The animals that were used in the present study were different than the mice that were used in the study by Jacobsen et al [46], but the preparation and instillation of the particle suspensions were carried out using the same protocol.

### Administration of carbon black

The young *apoE*^-/- ^mice were exposed to CB by i.t. instillation. One group received 0, 0.05, 0.5, 0.9 or 2.7 mg/kg bodyweight of CB and was sacrificed 24 hours later (n = 8-17). The second group received either 0 or 0.9 mg/kg bodyweight of CB and was sacrificed 2 hours later (n = 5-9) or two doses of 0 or 0.5 mg/kg bodyweight of CB 24 hours apart and was sacrificed 2 hours after the second exposure (n = 4-7). The aged mice received two doses of 0 or 0.5 mg/kg bodyweight of CB two weeks apart and were sacrificed 7 weeks after the first exposure (n = 11). After administration of CB, the mice were euthanized under general anaesthesia with Hypnorm-Dormicum by opening the thoracic cavity. The heart and aorta were carefully removed and placed in ice cold oxygenated physiological saline solution (PSS) and the surrounding connective tissue was removed.

We used the doses of CB in the range of 0.05-2.7 mg/kg because we have previously shown that exposures to other types of particulate matter, especially diesel exhaust particles, generate oxidatively damaged DNA in lung tissue by i.t. instillation or inhalation [60,61]. In addition, we used 0.5 mg/kg as daily dose because this dose had been used in a previous study on plaque progression in mice exposed to single walled carbon nanotubes [56]. In perspective, the daily dose of CB in the working environment can be as large as 0.12 mg/kg, assuming a threshold limiting value (TLV) for respirable CB of 2.5 mg/m^3^, 60% deposition, an average weight of a person of 70 kg, an 8 hour work day, and a respiratory volume of 0.7 m^3 ^pr. hour. The lowest of the applied doses was below this calculated value, and the remaining doses were increasingly above it.

To the best of our knowledge, no threshold limit value for CB in ambient air exists. However, the future air quality standard in the European Union for PM_2.5 _(25 μg/m^3^) indicates that the daily dose would be 3.6 μg/kg, which is more than one order of magnitude lower than the lowest dose in our study.

### Vasomotor function

The aorta from each of the young mice was cut in segments of approximately 1.5 mm in length starting from immediately after the three large side branches of the aortic arch. Three segments were obtained from each of the *apoE*^-/- ^mice exposed to the different concentrations of CB. The segments were mounted on steel pins with a diameter of 150 μm in the organ baths of the multi myograph 610M (Danish Myo Technology, Aarhus, DK) containing 5 ml cold oxygenated PSS continuously perfused with a 95% O_2 _and 5% CO_2 _gas mixture. The order in which the aorta segments were mounted in the organ baths was randomized with regard to exposure-group. The myograph was connected to a computer and the data were processed by the PC-program Myodaq (Danish Myo Technology, Aarhus, DK). The measurements of vasomotor function were carried out as previously described [18].

### Curve fitting and analysis

All steps in the concentration-response curves were recorded at the point considered to be the lowest (acetylcholine and sodium nitroprusside) or highest (phenylephrine and cocktail) steady state value obtained at that concentration of vasoactive reagent. The basal tone of the aorta was subtracted from all recordings of drug-induced vessel tone, estimated by placing a baseline in the Myodaq program. The relaxation caused by acetylcholine and sodium nitroprusside was expressed as the % relaxation of the precontraction tension produced by PGF_2_α. We used PGF_2_α because it induces a stable and reproducible vasocontraction that can be effectively relaxed with acetylcholine in the mouse aorta. The contraction caused by phenylephrine was expressed as the % of the maximal contraction obtained when stimulating the aorta segment with a cocktail consisting of K^+^-PSS, PGF_2_α and U46619. The EC_50 _and E_max _values were calculated using the GraphPad Prism version 4 (San Diego, CA, USA). The data were fitted to sigmoid curves with varying slopes using non-linear regression according to the following equation:

Y=Bottom+(Top−Bottom)/(1+10^((Log EC50−X)*HillSlope)

X is the logarithm of concentration and Y is the response. Y starts at Bottom and goes to Top with a sigmoid shape. The data points on each curve are expressed as mean ± SEM.

### Plaque progression

The aorta extending from the heart to the iliac arteries from each of the aged mice was cut longitudinally and placed with the intimal surface facing upwards on an objective glass with a few drops of PSS. The aorta was then carefully flattened by adding a cover slip while avoiding any folds or overlap of the vascular tissue. Digital images were obtained of the intimal surface of the aorta by means of an Olympus SZX7 stereo microscope and an Olympus Color View I camera. The level of atherosclerosis in the aorta was measured on the digital images with the public domain Java image processing program ImageJ.

### Immunohistochemistry

The heart and aortic root from each of the young mice and a segment of the brachiocephalic artery (BCA) from each of the aged mice were embedded in Tissue-Tek^® ^O.C.T.™ Compound (Sakura Finetek, Værløse, DK) and frozen on dry ice. Frozen 4 μm sections of the aortic root or BCA from the young or aged mice, respectively, were obtained on a CM3050 S-Cryostat (Leica Microsystems Nussloch GmbH, DE) and mounted on Super Frost + slides (Hounisen Laboratory Equipment, Risskov, DK). The mounted sections were fixed in 100% acetone and the endogenous peroxidase activity was blocked with Dako REAL™ Peroxidase-Blocking Solution (Dako, Glostrup, DK). ICAM-1 and VCAM-1 protein expression and the presence of 3-nitrotyrosine was visualized by means of biotin-conjugated antibodies, streptavidin horse radish peroxidase (HRP) (1:500) (Dako, Glostrup, DK) and 3,3'-diaminobenzidine (DAB) (1:50) (Dako, Glostrup, DK). The antibodies used were purified hamster anti-mouse CD54 (ICAM-1) monoclonal antibody (1:100) (BD Biosciences, Brøndby, DK) and biotin-conjugated mouse anti-hamster cocktail (1:100) (Biosciences, Brøndby, DK), biotin-conjugated rat anti-mouse CD106 (VCAM-1) monoclonal antibody (1:100) (BD Biosciences, Brøndby, DK) and biotin-conjugated goat anti-Nitro tyrosine polyclonal antibody (1:100) (Abcam, Cambridge, UK). Furthermore the sections were counterstained with Mayer's Haematoxylin. Finally the slides were fixed with cover slips using pertex mounting medium (Sakura Finetek, Værløse, DK). Sections of wild type rat spleen or aged *apoE*^-/- ^aorta, incubated with or without primary antibody, were used as positive and negative controls, respectively.

The slides were examined by means of an Olympus BX41TF-5 microscope and digital images were obtained with a ColorView I camera (Olympus Denmark A/S, Ballerup, DK) at 10× magnification.

The digital images were coded before being scored by an investigator who had not carried out the experiments and had no knowledge of which groups the depicted blood vessels belonged to. A semiquantative analysis of the images was carried out by visually scoring the intensity of the staining. The intensity of ICAM-1 and VCAM-1 staining in the young mice was scored as follows: 1) little staining, 2) few spots of staining in the endothelium, 3) strong staining of spots or long patches of staining in the endothelium, 4) strong staining of long patches of the endothelium. The blood vessels of the old mice were generally more intensively stained in both endothelium and intima; these images were scored according to the intensity in the endothelium. The 3-nitrotyrosine staining was scored as follows: 1) little staining, 2) slight staining near the endothelium, 3) staining in the intima, 4) strong staining in the intima. The images of at least 2-4 sections of blood vessels for each animal were scored and a mean score for each animal was calculated. The data are reported as the ranked intensity. Because there was overt difference in the staining of tissue from the young and old animals, the staining of the blood vessels from the respective ages were scored separately. The slides that were used for staining of ICAM-1, VCAM-1 and 3-nitrotyrosine were also used to determine the level of atherosclerosis in the BCA vessel by means of the program ImageJ.

### mRNA Expression

The mRNA was extracted from lung tissue samples using the TRIzol reagent (Invitrogen, Carlsbad, CA, USA). Approximately 50-100 μg frozen tissue from each sample was added to 1 ml TRIzol and homogenized. The samples were incubated at room temperature for 5 min before adding 0.2 ml chloroform and centrifuging at 12.000 rcf for 15 min. The supernatant was transferred to a new tube and the mRNA was precipitated with 0.5 ml isopropanol. The samples were incubated at room temperature for 10 min before centrifuging at 12.000 rcf for 10 min. The supernatant was discarded and the pellet was washed in ice cold 75% ethanol followed by centrifugation at 7.600 rcf for 5 min. The ethanol was discarded and the pellet was allowed to air dry shortly. The pellet was resuspended in 30 μl RNase free water by incubation at 55°C for 10 min.

The extracted mRNA samples were DNase treated using the RQ1 RNase-Free DNase kit (Promega, Madison, WI, USA). For each sample of 4 μl mRNA a stock solution containing 10 μl RNase free water, 4 μl DNase and 2 μl DNase buffer was added and the samples were incubated at 37°C for 30 min. 2 μl stop buffer was added and the samples were incubated at 65°C for 10 min.

The total mRNA was converted into cDNA by reverse transcriptase polymerase chain reaction by using the High Capacity cDNA Transcription Kit (Applied Biosystems, Calsbad, CA, USA). For each sample containing 150 ng mRNA in 10 μl, a stock solution containing 2 μl RT buffer, 0.8 μl dNTP mix, 2 μl Random Primers, 4.2 μl RNase free water and 1 μl Reverse Transcriptase was added. The samples were cycled at 25°C for 10 min, 37°C for 120 min, 85°C for 5 min and maintained at 4°C for a short period until storage at -20°C. A negative control with no Reverse Transcriptase was included in each run.

The quantification of gene expression was determined by real-time PCR using the Taqman^® ^gene expression assay. The primers used were: VCAM-1 (NM_011693.3), ICAM-1 (NM_010493.2), HO1 (NM_010442.2), MCP-1 (NM_011333.3) and as endogenous control: eukaryotic 18S rRNA (X03205.1), (Applied Biosystems). All samples were determined as triplicates of a stock solution containing 14.88 μl RNase free water, 18.75 μl Taqman^® ^Fast Universal PCR Master Mix (Applied Biosystems), 1.8 μl cDNA and 1.8 μl primer.

The standard curves for the primers were generated with 10 steps of 2× dilution starting from 10 μl cDNA.

The level of mRNA expression normalized to the level of 18S rRNA was calculated as 2^-ΔCt^. The relative mRNA levels in the exposure groups were standardized in order to obtain fold increases in mRNA level relative to the level in the control groups.

### Statistics

The data on vasomotor function were analyzed by ANOVA with unequal variance, as the exposure groups did not exhibit homogeneity of variance. The curve fittings were carried out in GraphPad Prism 4 and the E_max _and EC_50 _with corresponding 95% confidence intervals (CI) were calculated. Differences between groups with non-overlapping 95% CI were considered statistically significant at P < 0.05 level. The data on plaque progression, expression of adhesion molecules and presence of 3-nitrotyrosine, and expression of mRNA were assessed by non-parametric one-way ANOVA (Mann-Whitney U-test or Kruskall-Wallis test) with post-hoc Tukey-type multiple comparison test for effects showing statistical significance in the overall ANOVA test. Statistical significances were tested at P < 0.05 level. The differences in relative precontraction tension between exposure groups were analyzed by One-Way ANOVA (24 hours) or Two-Sample t-Test (2 hours and 24+2 hours). The association between relative precontraction tension and the maximal acetylcholine-induced vasorelaxation was assessed by Spearman correlation. The statistical analyses were performed in Statistica version 5.5 (StatSoft Inc., Tulsa, OK, USA) and SAS version 9.2 (SAS Institute Inc., Cary, NC, USA).

## Abbreviations

*apoE^-/-^*: apolipoprotein E knockout; Ach: acetylcholine; BCA: brachiocephalic artery; CB: carbon black; CI: confidence interval; DAB: 3,3'-diaminobenzidine; eNOS: endothelial nitric oxide synthase; HO-1: heme oxygenase 1; HRP: horse radish peroxidase; ICAM-1: inter-cellular adhesion molecule 1; i.t.: intrathracheal; MCP-1: macrophage/monocyte chemoattractant protein 1; NO: nitric oxide; PE: phenylephrine; PGF_2_α: prostaglandin F_2_α; PSS: physiological saline solution; SEM: standard error of the mean; SNP: sodium nitroprusside; VCAM-1: vascular cell adhesion molecule 1

## Competing interests

The authors declare that they have no competing interests.

## Authors' contributions

HWA, SL and PM conceived the study. LKV, HWA, SL and PM designed the experiments. LKV and JKF made the experiments on vasomotor function, assisted and supervised by MS. LKV made the experiments on plaque progression, immunohistochemistry and mRNA expression. The characterization of the particle suspensions and supervision of the animal experiments were carried out by NRJ. LKV made the draft of the manuscript, which was revised critically by SL and PM. All authors have read and approved the manuscript.
